# *Sigesbeckia glabrescens* Makino extract attenuated the collagen-induced arthritis through inhibiting the synovial hyperplasia and inflammation

**DOI:** 10.1186/s13020-020-00372-4

**Published:** 2020-08-27

**Authors:** Qiu Shuo Ma, Ke-Gang Linghu, Tian Zhang, Guan Ding Zhao, Wei Xiong, Shi Hang Xiong, Mingming Zhao, Wei Xu, Juan Yu, Hua Yu

**Affiliations:** 1 Institute of Chinese Medical Sciences, State Key Laboratory of Quality Research in Chinese Medicine, University of Macau, Room 8008, Building N22, Avenida da Universidade, Taipa, Macao, SAR China; 2 College of Pharmacy, Fujian University of Traditional Chinese Medicine, Fuzhou, Fujian China; 3 Zhangzhou Pientzehuang Pharmaceutical Ltd, Zhangzhou, Fujian China; 4 HKBU Shenzhen Research Center, Shenzhen, Guangdong China

**Keywords:** Rheumnnatoid arthritis, Synovial hyperplasia, Inflammation, *Sigesbeckia glabrescens* Makino, SW982, Nuclear transcription factor-kappa B

## Abstract

**Background:**

*Sigesbeckia glabrescens* Makino (SG) has been traditionally used for rheumatism and joint protection. However, the anti-arthritic effects and underling mechanisms of SG have not been demonstrated. In this study, we investigated the anti-arthritic effects and mechanisms of SG extract (SGE) on collagen-induced arthritic rats and interleukin (IL)-1β-stimulated human synovial SW982 cells.

**Methods:**

Rats were induced to arthritis by collagen for 15 days and then received SGE treatment for 35 days. The body weight and arthritis severity score of the rats were monitored weekly. At the end of the experiment, the radiographic and histological changes of rats’ hind paw were obtained; the serum C-reactive protein was detected by enzyme-linked immunosorbent assay (ELISA); the expression levels of interleukin (IL)- 1β, IL6 and IL-10 in the joint muscles were determined by ELISA and immunohistochemical staining; and the level of regulatory T cells (Tregs) in the spleen was detected using flow cytometry. In addition, 3-(4,5-Dimethylthiazol-2-yl)-2,5-diphenyltetrazolium bromide (MTT) assay and scratch wound healing assay were used to evaluate the proliferation of SW982 synovial cells. ELISA, western blot and immunofluorescence staining were used to investigate the anti-inflammatory mechanisms of SGE on IL-1β-induced SW982 cells and joint muscles of CIA rats.

**Results:**

SGE attenuated the collagen-induced hind paw swelling, cartilage damage and bone erosion. SGE inhibited the synovial hyperplasia to the articular cavity in the toe joint and ankle. Moreover, SGE decreased the production of C-reactive protein in serum and the expression of IL-6, IL-1β, cyclooxygenase-2 (COX-2) and phosphorylation of NF-κB p65 in the joint muscles. SGE also recovered the decreased Tregs. Results from the in vitro experiments showed that SGE not only inhibited the proliferation and migration of human synovial cell but also inhibited the IL-1β-induced expression of IL-6 and IL-8. Similarly, SGE inhibited the activation of NF-κB and the expression of COX-2.

**Conclusions:**

SGE attenuated the collagen-induced arthritis through inhibiting the synovial hyperplasia and inflammation.
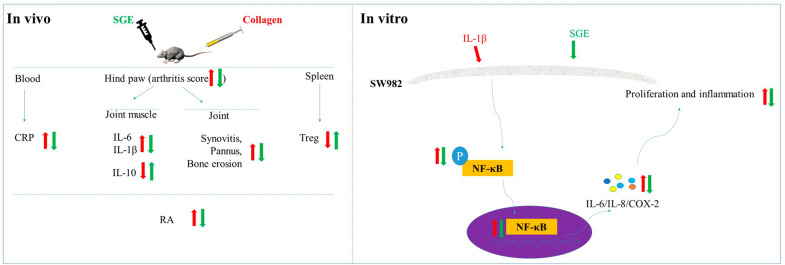

## Background

Rheumatoid arthritis (RA) is an autoimmune disease characterized by chronic inflammation, synovial tissue proliferation, cartilage damaging and bone erosion. Almost all RA patients lost the joint function finally, which highly affects the life quality of the patients, and leads to serious social problems and a tremendous economic burden [1].

Etiology and the pathogenesis of RA are complex, referring to various types of cells such as macrophages, T/B cells and fibroblasts [2]. As an important component of synovial tissue, the synovial fibroblasts, also called fibroblast-like synoviocytes (FLS), play critical role during the progress of joint destruction through secreting various cytokines, proteases and arachidonic acid metabolites [3]. Activation of FLS promotes the clinical symptoms and the process of RA [3]. Excessive proliferation and invasion of FLS have been reported to be involved in the pathogenesis of RA. Blocking the activation of FLS to reduce the production of cytokines has become a promising RA therapy [4]. Therefore, FLS becomes a critical target cell for studying the treatment and pathogenesis of RA.

Sigesbeckiae Herba (SH), a traditional anti-inflammatory herbal medicine, had been used for rheumatism from Tang dynasty in China [5]. Currently, the officially authorized plant origins for SH include *Sigesbeckia pubescens* Makino (SP), *S. glabrescens* Makino (SG), and *S. orientalis* L. (SO). SO had been reported to attenuate λ-carrageenan-induced paw edema and LPS-induced systemic inflammation in mice [6]. SP had shown therapeutic effect on collagenase-induced osteoarthritis by inhibiting cartilage damaging in rabbits [7]. Although there are growing number of reports about the pharmacological properties of SG in vitro, such as anti-inflammation [8], immunomodulation [9], and anti-tumor [10], it has not been demonstrated about the pharmacological properties of SG on arthritis in vivo. In this study, we investigated the therapeutic effects of SG in collagen-induced arthritic rats, and further revealed the anti-proliferation and anti-inflammatory mechanisms of SG on interleukin -1β-stimulated human synovial SW982 cells. Our work suggests the therapeutic effects of SG on RA treatment, and provides better understanding for rational applications of SP, SO and SG in clinics.

## Methods

The Minimum Standards of Reporting Checklist contains details of the experimental design, and statistics, and resources used in this study (Additional file 1).

### Chemicals and reagents

3-[4, 5-Dimethyl-2-thiazolyl]-2, 5-diphenyltetrazolium bromide (MTT), dimethyl sulfoxide (DMSO), indomethacin (IND) and lipopolysaccharides (LPS, *Escherichia coli* O111:B4) were purchased from Sigma-Aldrich (St. Louis, MO, United States). FITC anti-rat CD4, PE anti-rat CD25, Alexa Fluor^®^ 647 anti-mouse/rat/human FOXP3, (Alexa Fluor^®^ 647, PE and FITC) Mouse IgG1, κ isotype ctrl and True-Nuclear™ Transcription Factor Buffer Set were obtained from Biolegend (San Diego, CA, USA). Fetal bovine serum (FBS), 0.25% Trypsin–EDTA (w/v), dulbecco’s modified eagle’s medium (DMEM), penicillin–streptomycin (10,000 U/mL, P/S) and phosphate-buffered saline (PBS) were purchased from Thermo Fisher Scientific (Waltham, MA, USA). Interleukin (IL)-1β protein and enzyme-linked immunosorbent assay (ELISA) kits were supplied by Neobioscience Technology Co., Ltd. (Shenzhen, China). Safranin O, Fast Green FCF and Streptavidin Peroxidase immunohistochemistry kit were obtained from Solarbio (Beijing, China). Primary antibodies against IL-1β, IL-6 and IL-10 were purchased from Beyotime Biotechnology (Shanghai, China) and were diluted with 1:100, 1:100, and 1:50 respectively. Primary antibodies against NF-κB p65, phosphorylation (P)-p65, COX-2 and GAPDH (glyceraldehyde-3-phosphate dehydrogenase) and the secondary antibody were purchased from Cell Signaling Technology (Danvers, MA, United States), all antibodies were diluted with 1:1000.

### Herbs and herbal extracts

The herb of *Sigesbeckia glabrescens* Makino (SG) was collected from Jinyun (Zhejiang province, China; longitude: 120.61, latitude: 28.66) and authenticated by Dr. Hua Yu (the corresponding author). The voucher specimen (No. SG-03) was deposited at the Institute of Chinese Medical Sciences, University of Macau, Macao SAR, China.

The SG extract (SGE) used in this study is a part of the extract prepared previously using the herbal material of SG-03 [11]. The contents of five components in the SGE were determined to be 4.37% (kirenol), 6.30% (3-*O*-methylquercetin), 1.09% (3,7-dimethyl-5,3′,4′-trihydroxyflavone), 0.43% (darutoside) and 1.02% (Lecocarpinolide B), respectively, by UPLC-PDA analysis.

### Experimental animals and treatments

Male Wistar rats (7–8 weeks old) were fed on a standard animal laboratory environment (free access to water and food, 20–22 °C, relative humidity of 50% and 12-h light/dark cycle). All the experimental protocols were in accordance with the National Institutes of Health guidelines for the Care of Use of Laboratory Animals and approved by the Animal Research Ethics Committee (reference No: UMARE-029-2016), University of Macau, Macao SAR, China.

The model of collagen-induced arthritis (CIA) in rats was established using emulsified bovine type II collagen in incomplete Freund’s adjuvant according to the protocol of manufacture (Chondrex, Inc., NE Redmond, WA 98052). Rats were randomly divided into 6 groups according to body weight (n = 6 in each group) as following: Ctrl (Vehicle), CIA, CIA-SGE (0.19, 0.95 or 1.91 g/kg) and indomethacin (2.5 mg/kg). All drugs were intragastrically administered daily for 35 consecutive days. The body weight was recorded every week. Clinical scores of arthritis were evaluated every week as previously described [12]. At the end of the experiment, blood samples were collected from rat orbit for ELISA assay. The rats were sacrificed by CO_2_ inhalation, tissues or organs were isolated under ice-cold for indicated experiments.

### ELISA assay for serum and joint muscles

The joint muscles were lysed by cell lysis buffer (Beyotime, Jiangsu, China) and the concentration of lysates were determined using a BCA protein assay kit (Thermo Fisher Scientific, MA, USA). C-reactive protein in the serum as well as the inflammation-related proteins of IL-6, IL-1β and IL-10 in the lysates were determined with ELISA kits in accordance with the manufacturer’s protocol.

### Flow cytometry analysis

The spleen was grinded and filtered by a cell strainer with 40 μm nylon to make single-cell suspensions. Then the red blood cells were excluded by red blood cell lysate. The purified splenocytes (5–10 × 10^5^ cells) were surface-stained with anti-rat CD4-FITC/CD25-PE for 30 min on ice in darkness, then permeabilized and stained with anti-mouse/rat/human Foxp3-Alexa Fluor^®^ 647 for another 30 min. The acquisition of flow cytometry data and the analysis of a cell population of at least 1 × 10^4^ splenocytes were performed on flow cytometer with the BD FACSDiva Software (FACSCanto, BD Biosciences).

### Radiography, immunohistochemistry, histopathology and proteoglycan evaluation

On day 50, plain radiographs of the hind paws were obtained using an IVIS Lumina XR Imaging System (Caliper, MA, USA). Then the bone and muscle were separated into two parts and fixed in 10% neutral phosphate-buffered formalin respectively. 24 h later, the muscles were used to detect the expression levels of IL-6, IL-1β and IL-10 with immunohistochemical staining as previously [13]; 7 days later, the bone specimens were decalcified in mixed acid solution (8% hydrochloric acid, v/v; 5% acetic acid, v/v; 10% salicylic acid, m/v) for 2–3 weeks. The decalcified bones were used for histopathological evaluation on the joint injury with hematoxylin & eosin staining and proteoglycan assessment on cartilage degeneration with Safranin O staining as previously [13, 14].

### Culture of human synovial cell

The human synovial SW982 cell line was obtained from the American Type Culture Collection (ATCC; Manassas, VA, USA). The cells were cultured in a flask (25 cm ^2^; Thermo Fisher Scientific, MA, USA) with 10% FBS and 1% P/S in the atmosphere of 95% humidity and 5% CO_2_ at 37 °C. Cells were sub-cultured after trypsinization when they had grown to complete confluence.

### Cell viability and ELISA assay

SW982 cells were seeded in 96-well plates at a density of 1 × 10^4^ cells/well, 24 h later, cells were co-treated with the indicated concentrations of SGE for another 24 h in the absence or presence of IL-1β (20 ng/mL). Thereafter, the cytokines of IL-6 and IL-8 in the culture medium were quantified using an ELISA kit according to the manufacturer’s instructions, cells in the plate were used to assay the cell viability in accordance with our previous methods [11].

### Scratch wound healing assay

SW982 cells were seeded in 24-well plates (1 × 10^5^ cells/well) and allowed to form a monolayer cell. A scratch wound was built in each group with a 10 μL pipette tip. Washing to discard the unattached cells with PBS, obtaining the scratch wound by taking photos with the microscope. Then cells were co-treated with the SGE (50, 100, 200 μg/mL) in the absence or presence of IL-1β (20 ng/mL), 24 h later, the scratch wound was obtained by taking photos under the microscope. The scratch wound size was determined with Image J software.

### Western blot analysis

To investigate the effects of SGE on NF-κB p65, P-p65 and COX-2, SW982 cells were seeded in 6-well plates at a density of 5 × 10^5^ cells/well, 24 h later, cells were pretreated with the SGE (50, 100, 200 μg/mL) for 1 h then stimulated with IL-1β (20 ng/mL) for 12 h; the joint muscles were separated from the collected hind paw of mice and lysed by cell lysis buffer. The protocols of western blot experiments and results analysis were operated as previously [11].

### Immunofluorescence staining

SW982 cells were seeded into a confocal culture dish (NEST Biotechnology Co., Ltd; Jiangsu, China) at a density of 5 × 10^5^ cells per dish. 24 h later, the cells were pre-treated with SGE for 1 h then stimulated with IL-1β (20 ng/mL) for 1 h. Then the cell staining was conducted as previously [11].

### Statistical analysis

All data from a minimum three experiments were presented as mean ± SD. Data were analyzed on GraphPad Prism 6.0 software based on a one-way ANOVA with Dunnet’s multiple comparisons test; *P *< 0.05 was considered difference significantly.

## Results

### The effects of SGE on body weight and clinical score of arthritis index

**Fig. 1:**
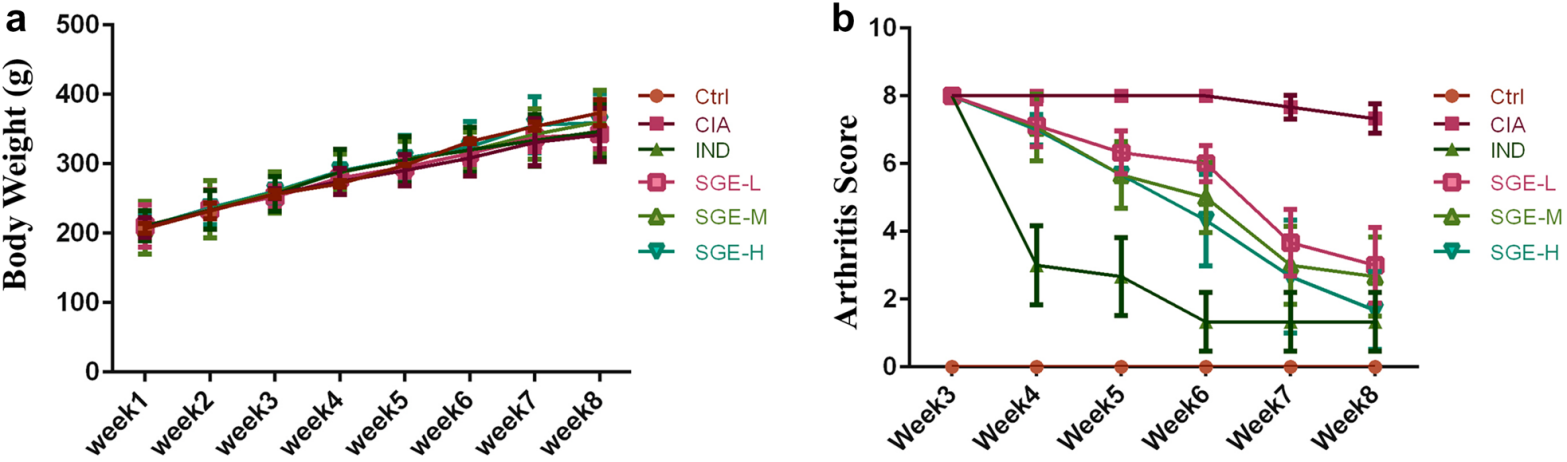
The effects of SGE on body weight and clinical score of arthritis index. The 36 Wistar male rats were randomly divided into 6 groups according to body weight (*n *= 6 in each group) as following: Ctrl (Vehicle), CIA (collagen-induced arthritis), CIA- SGE (SG extract, 0.19, 0.95 and 1.91 g/kg) and CIA-IND (indomethacin, 2.5 mg/kg). After the CIA was reproduced, drugs were administered intragastrically daily for 35 consecutive days. The body weight was recorded every week. Clinical arthritis scores were evaluated once a week. The growth curve of rats (*n *= 6) (**a**); The severity of arthritis by the arthritis score (*n *= 6) (**b**)

As shown in Fig. 1a, no significant differences on the growth curves among the Ctrl (control) and SGE-treated groups were observed, indicating the safety of all doses of SGE. As shown in Fig. 1b, at the third week after collagen induction, the joint score of the rats reached to the maximum (8 points). After treatment with IND (indomethacin, positive drug) or different dosages of SGE, the joint score was gradually decreased with the therapeutic period, while the model group showed no significant changes.

### SGE moderated the inflammation-related proteins in serum and joint muscles

**Fig. 2:**
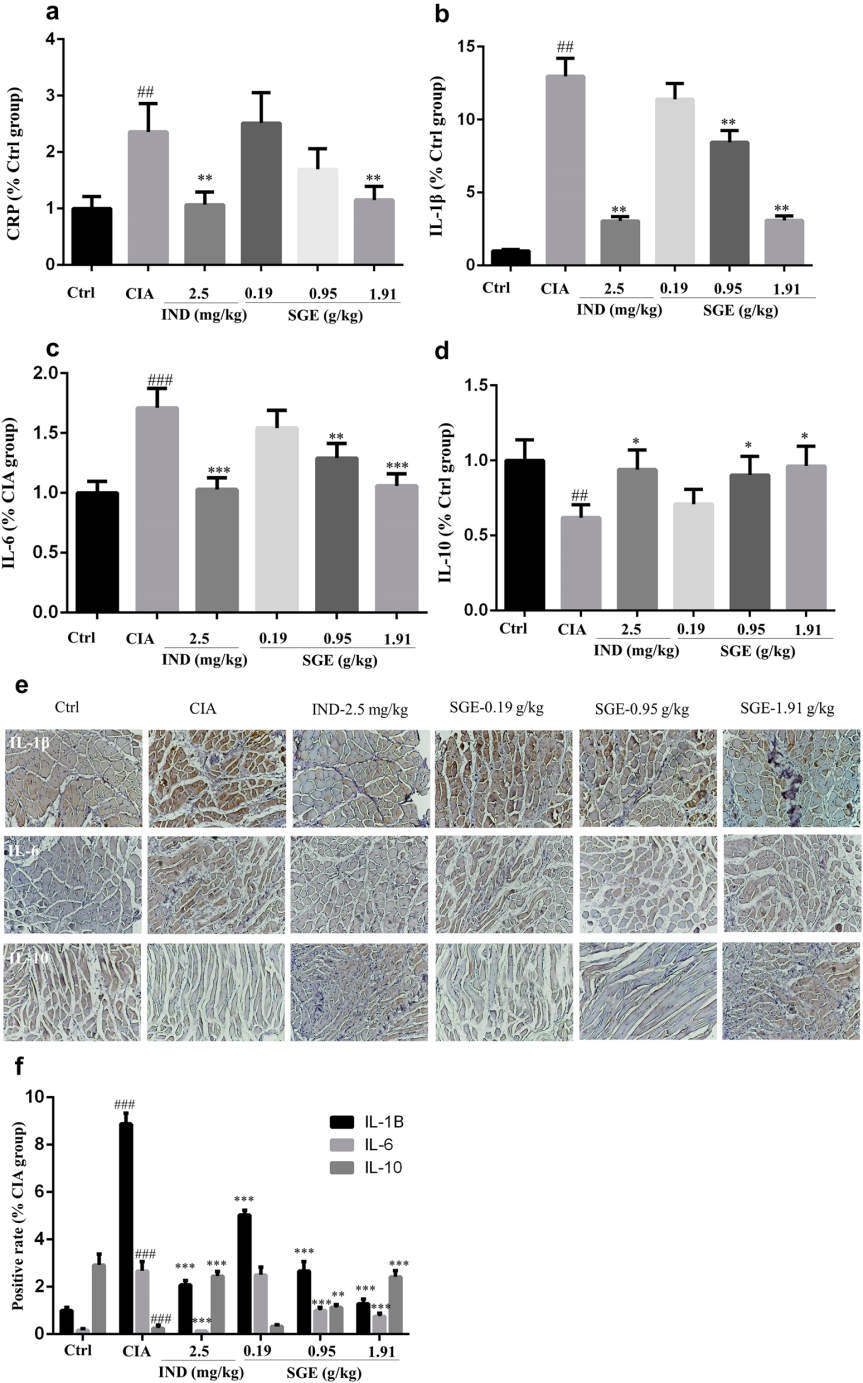
SGE moderated the inflammation-related proteins in serum and the joint muscles. On the day-50, the blood and joint muscles were collected. The serum C-reactive protein (CRP) (**a**) was detected by ELISA kit. The expression levels of IL-1β (**b**), IL-6 (**c**) and IL-10 (**d**) in joint muscles were determined with ELISA kits and immunohistochemical staining (E&F). (^#^*P *< 0.05, ^##^*P *< 0.01 and ^###^*P *< 0.001 vs. Ctrl group; ^*^P < 0.05, ^**^P < 0.01 and ^***^P < 0.001 vs. CIA group; *n *= 6)

As shown in Fig. 2a, the expression of serum c-reactive protein was increased in CIA group, which could be reduced by SGE dose-dependently, also decreased by IND. Additionally, SGE decreased the pro-inflammatory proteins of IL-1β (2B&F) and IL-6 (2C&F) and increased the anti-inflammatory protein of IL-10 (2D&F) in the joint muscles. The therapeutic effects of SGE (1.91 g/kg) were comparable to those of IND (2.5 mg/kg).

### SGE upregulated the level of CD25^+^CD4^+^FOXP3^+^ cells in spleen

**Fig. 3:**
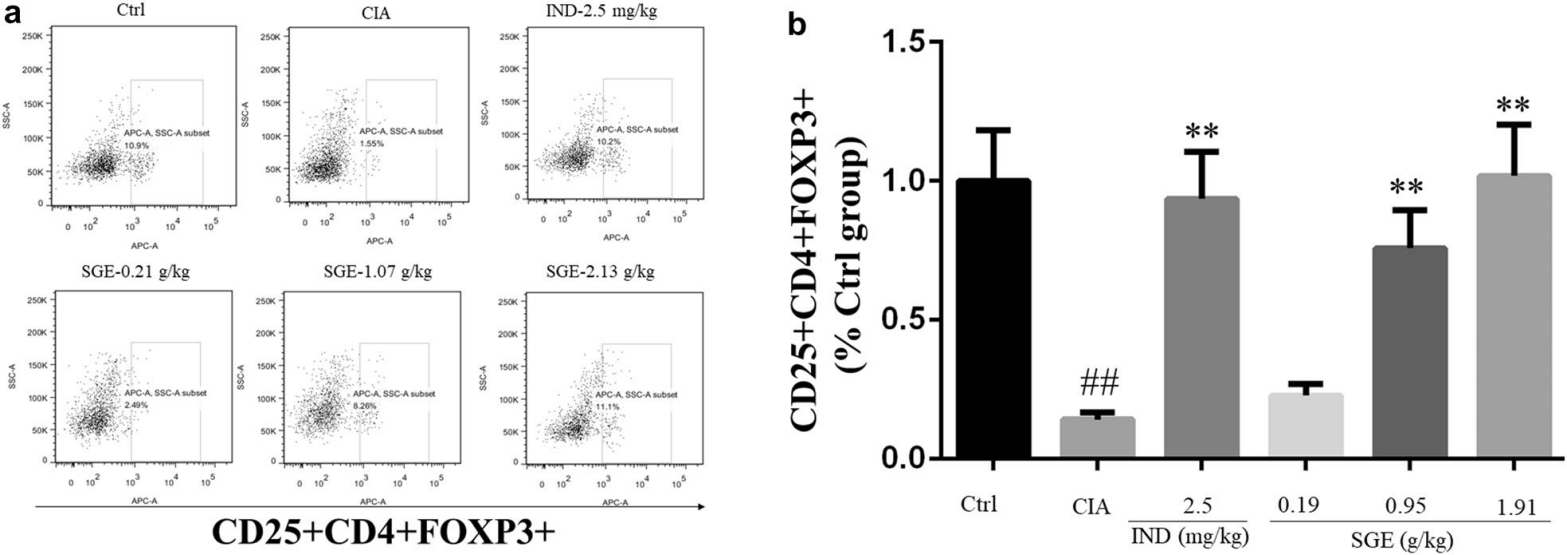
SGE upregulated the level of CD25^+^CD4^+^FOXP3^+^ cells in spleen. The purified splenocytes (5 ~ 10 × 10^5^ cells) were surface-stained with anti-rat CD4-FITC/CD25-PE for 30 min on ice in darkness, then permeabilized and stained with anti-mouse/rat/human Foxp3-Alexa Fluor^®^ 647 for another 30 min. The acquisition of flow cytometry data and the analysis of a cell population of at least 1 × 10^4^ splenocytes were performed on flow cytometer with the BD FACSDiva Software (FACSCanto, BD Biosciences). Representative images of flow cytometry for CD4^+^CD25^+^Foxp3^+^ regulatory T cells (**a**). Quantification of the number of CD4^+^CD25^+^Foxp3^+^ regulatory T cells (**b**). (^##^*P *< 0.01 vs. Ctrl group; ^**^P < 0.01 vs. CIA group; *n *= 6)

As shown in Fig. 3a, the percentages of regulatory T cells (Tregs) with CD25^+^CD4^+^FOXP3^+^ in the splenocytes of the Ctrl, CIA, CIA + IND, CIA + SGE (0.19 g/kg), CIA + SGE (0.95 g/kg) and CIA + SGE (1.91 g/kg) groups were 11.02 ± 1.63%, 1.57 ± 0.23%, 10.31 ± 1.53%, 2.52 ± 0.37%, 8.35 ± 1.24% and 11.22 ± 1.66% respectively. The percentage (Ctrl group  %) of Tregs in each group was shown in Fig. 3b.

### SGE alleviated the radiologic and pathological characteristics of hind paw joints

**Fig. 4:**
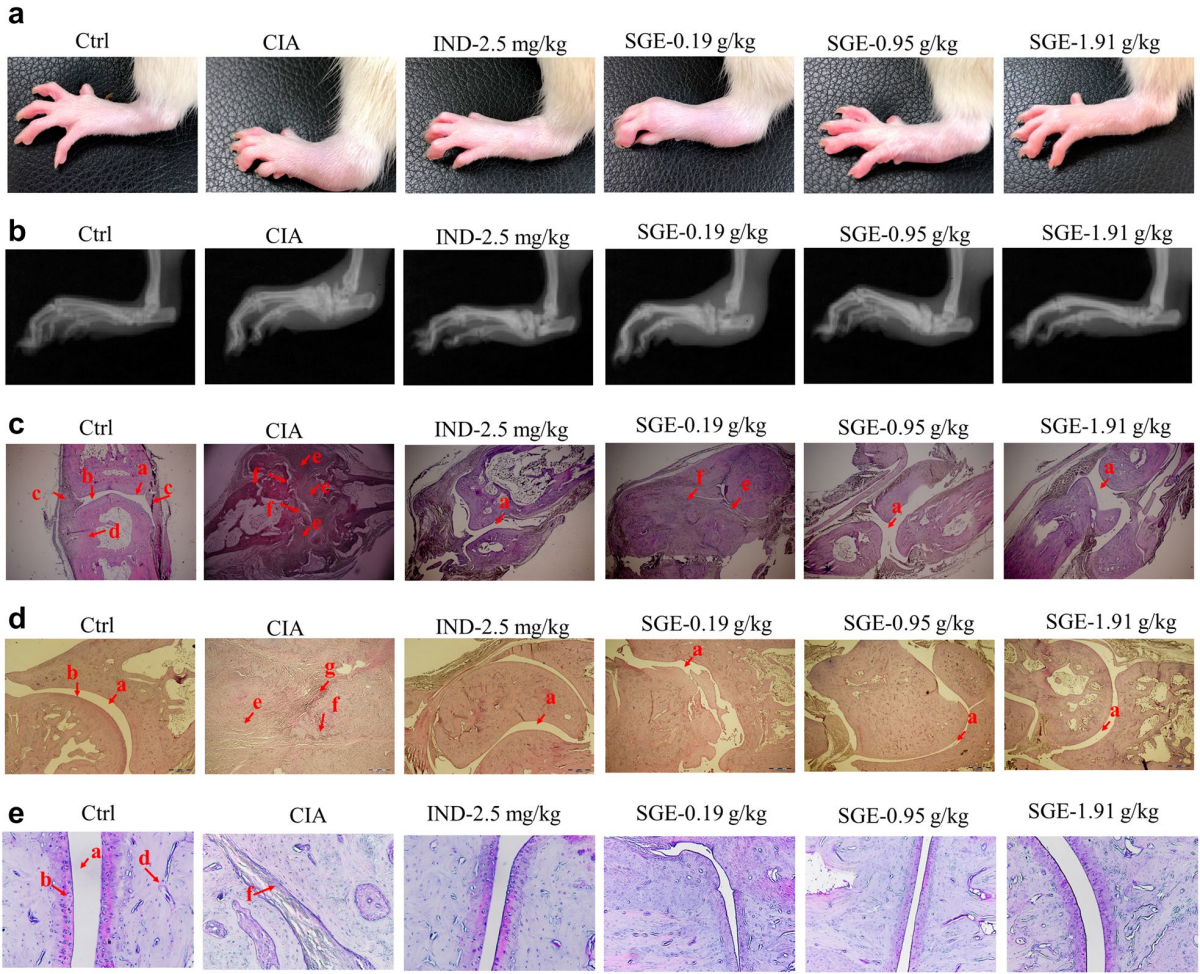
SGE alleviated the radiologic and pathological characteristics of hind paw joints. Photographs (**a**) and X-ray images (**b**) of the hind paws on day-50 after the first collagen immunization. Histopathological evaluation on the injury of toe joints (**c**) and ankle joints (**d**) with hematoxylin & eosin staining. Proteoglycan assessment on cartilage degeneration with Safranin O staining (**e**). (a, articular cavity; b, cartilage; c, synovial membrane; d, bone; e, pannus; f, bone erosion; g, inflammatory infiltration)

As shown in Fig. 4a, the hind paw swelling was obviously observed in the CIA group, which could be attenuated by the intake of SGE or IND. X-ray images (Fig. 4b) showed that the joint and bone structures were damaged seriously in the CIA group, which could be attenuated by treating with SGE or IND. We further evaluated the histopathological characterizations of toe joint and ankle in Fig. 4c–e. In the Ctrl group, both the toe joint and ankle showed a complete and normal articular cavity. However, in the CIA group, an obvious synovial hyperplasia to the articular cavity (formation of pannus) in the toe joint and ankle was observed, which led to the narrow articular cavity, cartilage degeneration &defect, and bone erosion. Among the drug treatment groups, synovial cell proliferation was alleviated, the number of infiltrated inflammatory cells was significantly decreased, and bone or cartilage damage was significantly reduced.

### SGE alleviated IL-1β-induced proliferation and migration in the SW982 human synovial cell line

**Fig. 5:**
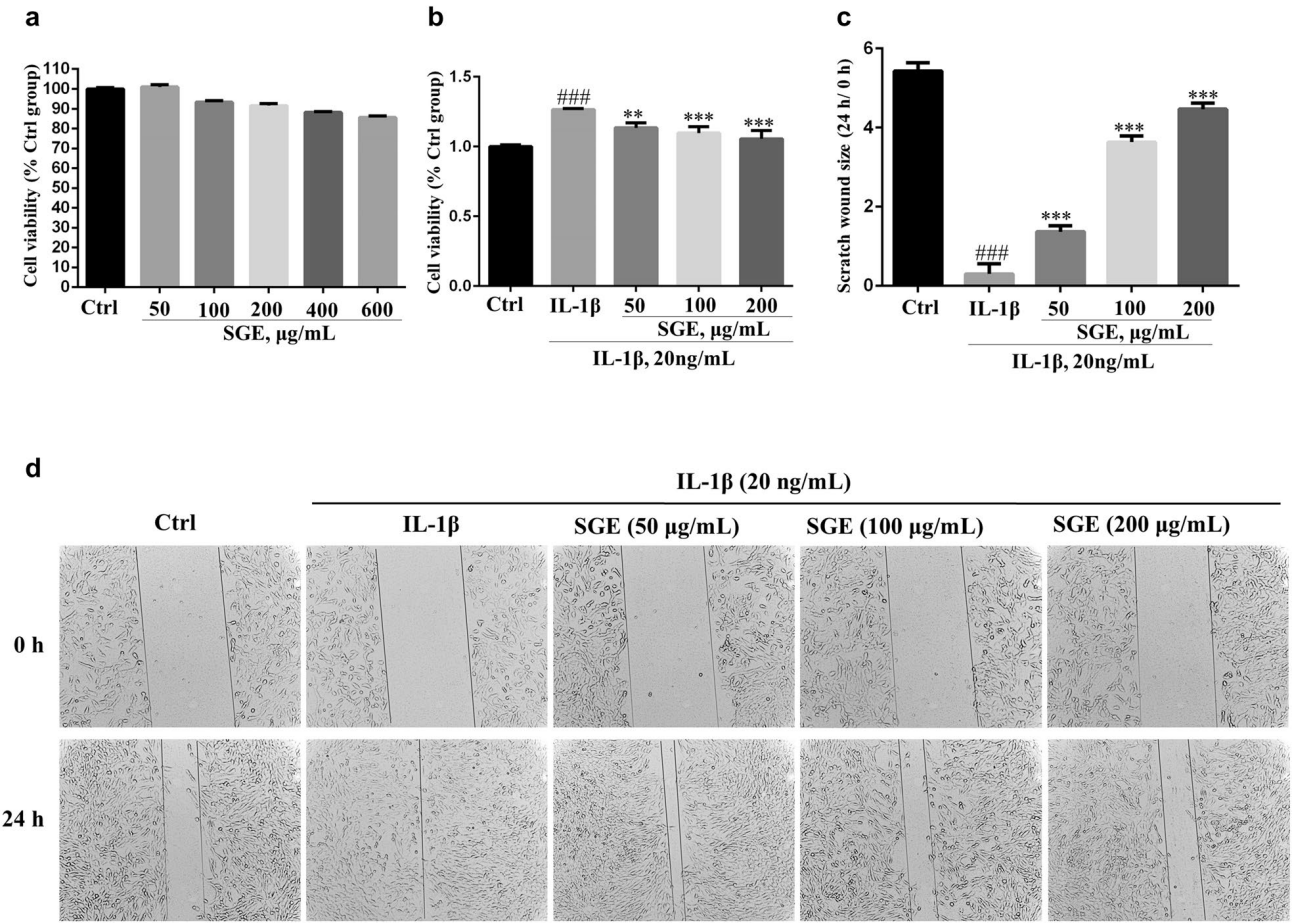
SGE alleviated IL-1β-induced proliferation and migration in the human synovial SW982 cell line. SW982 cells were seeded in 96-well (1 × 10^4^ cells/well) or 24-well (1 × 10^5^ cells/well) plates and allowed to grow for 24 h. A scratch wound was built in each group with a 10 μL pipette tip in the 24-well plate. Cells were co-treated with the indicated concentrations of SGE for 24 h in the absence or presence of IL-1β (20 ng/mL). The cell viability was determined by MTT assay (**a**, **b**). The images of the scratch wound were obtained with a microscope (**d**) and scratch wound changes were analyzed (**c**) after determining the wound size with Image J software (^#^*P *< 0.05, ^###^*P *< 0.001 vs. Ctrl group; ^**^P < 0.01, ^***^P < 0.01 vs. IL-1β group; *n *= 3)

The MTT results showed that SGE was not obvious toxic to SW982 cells when the concentrations of SGE was less than 600 μg/mL for 24 h (Fig. 5a), and the SGE (50, 100, 200 μg/mL) alleviated the IL-1β-induced cell proliferation when co-incubation the cells with IL-1β (20 ng/mL) and SGE for 24 h (Fig. 5b). Besides, SGE (50, 100, 200 μg/mL) inhibited the cells migration in IL-1β-induced SW982 cells (Fig. 5c, d).

### SGE reduced the production of IL-6 and IL-8 in SW982 cells

**Fig. 6:**
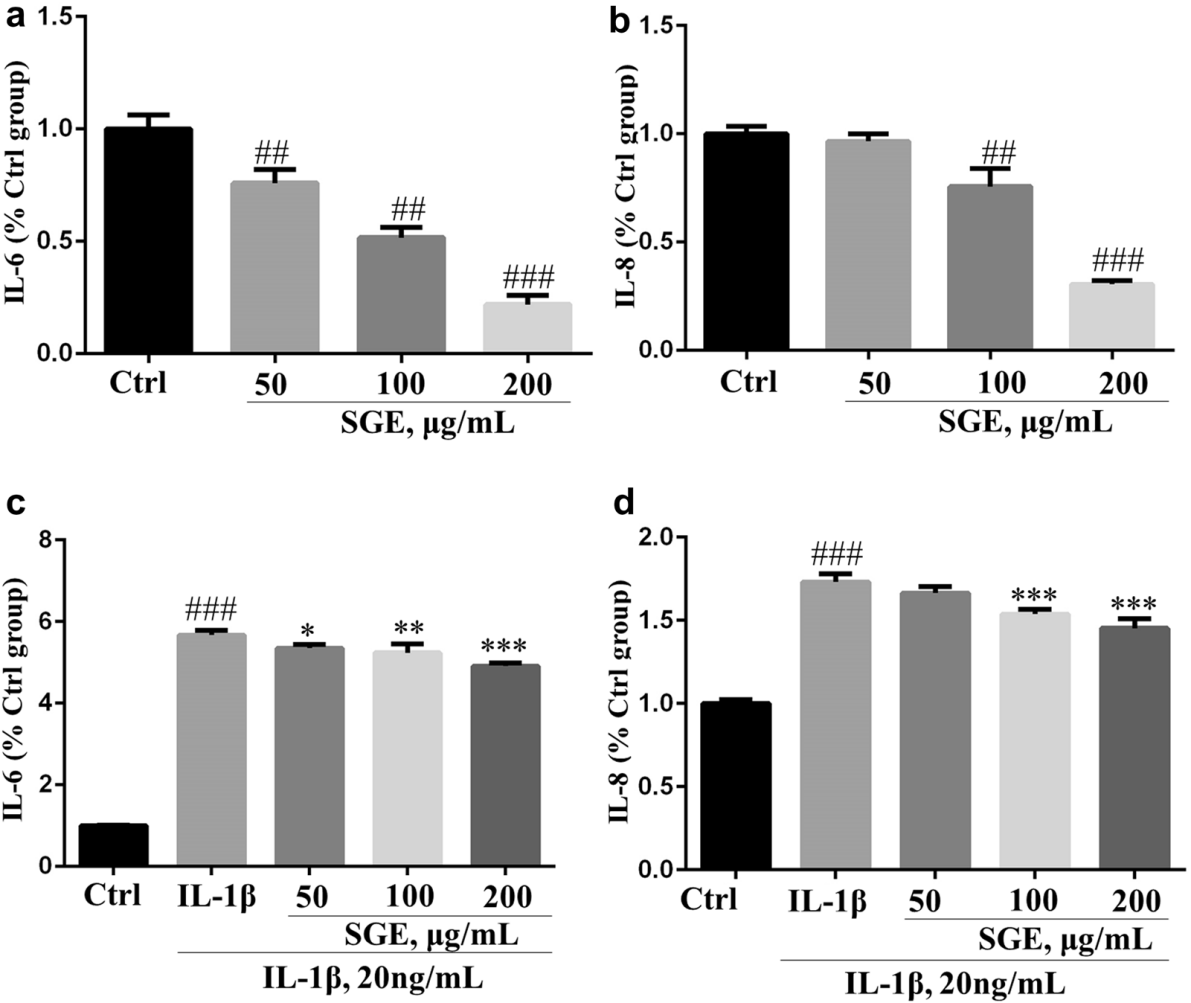
SGE reduced the production of IL-6 and IL-8 in SW982 cells. SW982 cells were seeded in 96-well plates (1 × 10^4^ cells/well) and allowed to grow for 24 h. Cells were co-treated with the indicated concentrations of SGE for 24 h in the absence or presence of interleukin (IL)-1β (20 ng/mL). Thereafter, the cytokines of IL-6 (**a**, **c**) and IL-8 (**b**, **d**) in the supernatant was quantified using an ELISA kit according to the manufacturer’s instructions. (^##^*P *< 0.01, ^###^*P *< 0.001 vs. Ctrl group; ^*^P < 0.05, ^**^P < 0.01, ^***^P < 0.001 vs. IL-1β group; *n *= 3)

As shown in Fig. 6c, d, IL-1β induced the increase of IL-6 and IL-8, which could be reduced by SGE dose-dependently. Additionally, SGE also reduced the basal level of IL-6 and IL-8 in SW982 cells when co-treatment with SW982 cells for 24 h in the absence of IL-1β (Fig. 6a, b).

### SGE suppressed the activation of NF-κB p65 and the expression of COX-2 in IL-1β-induced SW982 cells and CIA rats

**Fig. 7:**
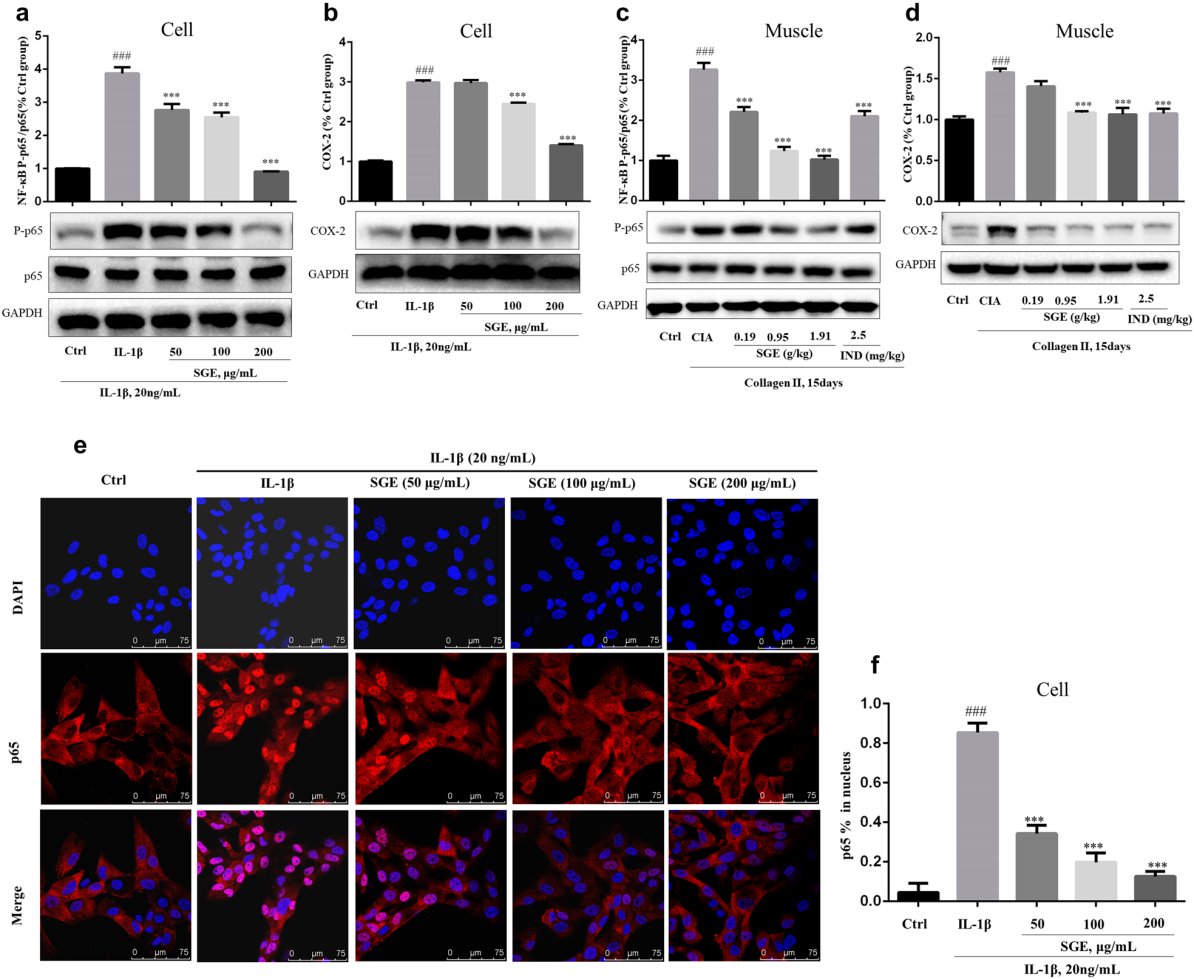
SGE blocked the activation of NF-κB in IL-1β-induced SW982 cells and joint muscles of CIA rats. SW982 cells were seeded in 6-well plates and confocal dish (5 × 10^5^ cells/well) and allowed to grow for 24 h. Cells were pretreated with the indicated concentrations of SGE for 1 h, then stimulated with IL-1β (20 ng/mL) for 1 h or 12 h; on day 50, the joint muscles were separated from the hind paw of mice and lysed by cell lysis buffer. The expression of P-p65 and COX-2 in SW982 cells (**a**, **b**) and joint muscles (**c**, **d**) were detected by western-blotting. The translocation of p65 (**e**, **f**) was determined by immunofluorescence staining (^###^*P *< 0.001 vs. Ctrl group; ^***^P < 0.001 vs. IL-1β group or CIA group; *n *= 3 for the cell experiment and *n *= 6 for the animal experiment)

NF-κB is a critical signaling pathway involved in the development of RA, the phosphorylation of NF-κB p65 (P-p65) enhanced the NF-κB p65 to enter the nucleus to mediate the transcription of inflammatory genes. As shown in Fig. 7, IL-1β increased the expression levels of P-p65 (Fig. 7a) and promoted the translocation of p65 from cytoplasm to nucleus (Fig. 7e, f) in SW982 cells, which could be reduced dose-dependently by the treatment of SGE. The inhibition of NF-κB by SGE also inhibited the expression of COX-2 (Fig. 7b) which was a critical protein involved in inflammation. Similarly, the SGE treatment reduced the expression levels of P-p65 (Fig. 7c) and COX-2 (Fig. 7d) in the joint muscle of CIA rats.

## Discussion

Rheumatoid arthritis (RA) is a kind of chronic inflammatory disease accompanied by joint damage and bone erosion, serious disabling risk of RA arose huge economic burden to the society. The development of effective strategies for the prevention and therapy of RA is highly desired [15].

*Sigesbeckia glabrescens* Makino (SG) is one of the plant origins for Sigesbeckiae Herba (SH) which has been traditionally used for rheumatism [5]. Although SG was used as SH with other two plants (SP and SO), there is lack of the in vivo evaluation for SG. Our previous study showed that SG attenuated the LPS-induced inflammation on macrophages significantly [11], which indicated the potential of SG on RA therapy. An additional therapeutic and mechanical demonstration of SG on RA animal model is essential to contribute the rational use of this herb medicine and shorten the gap between the clinic application and basis research.

In the present study, we focused the investigation on the anti-arthritic effects of SG extract (SGE) in rats with collagen-induced arthritis (CIA). As shown in Fig. 1a, the growth of rats between the control and SGE-treated group was no difference, which indicated that SGE was non-toxicity to rats. Moreover, SGE attenuated the CIA with the reduced clinical scores (Fig. 1b).

The pathological mechanisms of RA had been widely investigated and reported [16]. Production of unnormal autoantibodies usually lead to the activation of T lymphocytes and B lymphocytes, which further interact with the macrophages to produce various inflammation-related cytokines. These cytokines could act on synovial membrane and cartilage, initiating excessive proliferation and invasion of synovial fibroblasts to the articular cavity, thus finally leading to joint damage and bone erosion. Therefore, the continuous dysfunction of immune system and the local joint inflammation would be the key causes. CD4^+^CD25^+^FOXP3^+^ T lymphocytes are the critical Tregs to maintain the function of immune system. Lu et al. reported that stimulation of the CD4^+^CD25^+^Foxp3^+^ would alter potent antiarthritic effect against CIA [17]. Our results show that the proportion of CD4^+^CD25^+^FOXP3^+^ T lymphocytes in the spleen of CIA rat was much lower than vehicle rats, but the SGE could upregulate the proportion of Tregs (Fig. 3a, b). C-reactive protein (CRP) is a blood plasma protein, whose circulating concentrations rise in response to inflammation [18]. CRP is critical inflammatory marker dramatically increased in the peripheral blood RA patients [19]. As shown in Fig. 2a, the results showed that SGE reduced the increased CRP in CIA rats, which correlated with the rebalanced immune system (Fig. 3a, b). As shown in Fig. 4a, results showed that SGE ameliorated the hind paw swelling induced by collagen, which were consistent with the decreased expression of IL-6 & IL-1β and increased IL-10 in the inflammatory joint muscles (Fig. 2b–f). Additionally, after treatment with SGE, synovial cell proliferation was alleviated, the number of infiltrated inflammatory cells was significantly decreased, and the bone or cartilage damage was significantly reduced (4b–e).

The significant inhibition of SGE on synovial hyperplasia and inflammation on rats inspired us a further investigation into the therapeutic effects and mechanisms on the human fibroblast-like synoviocytes. The best-known cell model used to study synovitis in RA is the human synovial sarcoma cell line (SW982) [20]. Hence, we validated the effects and mechanisms of SGE on human SW982 synovial cell.

The transcription factor NF-κB, a typical signal pathway involved in inflammation and proliferation, has been well recognized as a pivotal regulator of RA [21]. In our previous research, SGE inhibited the activation of NF-κB on RAW264.7 cell to attenuate inflammation [11, 22]. As shown in Fig. 7a, e, f, SGE also inhibited the activation of NF-κB on IL-1β-induced SW982 cells. Further investigations suggested SGE reduced the expression of pro-inflammatory cytokines and protein (Fig. 6a–d, and Fig. 7b) as well as the proliferation (Fig. 5b) and migration (Fig. 5c, d) in IL-1β-induced SW982. Similarly, the SGE treatment inhibited the activation of NF-κB (Fig. 7c) and the production of IL-1β,IL-6 and COX-2 (Fig. 2b–f, and Fig. 7d) in the joint muscle of CIA rats.

It has been evidenced that NF-κB is involved in abnormal apoptosis and proliferation of RA fibroblast-like synovial cells [23]. In RA, NF-κB is over-expressed in the inflamed synovium [24], where its activity may enhance recruitment of inflammatory cells and production of proinflammatory mediators such as IL-1β, IL-6, IL-8 and TNF-α [25]. Therefore, SGE might attenuate the RA through inhibiting the synovial hyperplasia and inflammation by blocking NF-κB signal.

## Conclusions

In summary, results from the in vivo and in vitro suggests the therapeutic effects of SG on RA, and the mechanisms would be the inhibition of synovial hyperplasia and inflammation through blocking NF-κB signaling and balancing immune system. In accordance with the previous in vivo reports showing the cartilage protection of SP [7], and the amelioration to paw edema by SO [6], our work further provided in vivo evidence for SG on RA treatment therefore suggesting the similarities in pharmacology and therapeutic effectiveness of the three species of SH (SP, SO, SG) in treating RA.

## Data Availability

The datasets used and/or analyzed during the current study are available from the corresponding author on reasonable request.

## Abbreviations

RA: Rheumatoid arthritis
SGE: SG extract
MTT: 3-(4,5-Dimethylthiazol-2-yl)-2,5-diphenyltetrazolium bromide
ELISA: Enzyme-linked immunosorbent assay
Tregs: Regulatory T cells
COX-2: Cyclooxygenase-2
FLS: Fibroblast-like synoviocytes
SH: Sigesbeckiae Herba
SP: *Sigesbeckia pubescens* Makino
SG: *Sigesbeckia glabrescens* Makino
SO: *Sigesbeckia orientalis* L
DMSO: Dimethyl sulfoxide
DMEM: Dulbecco’s modified eagle’s medium
IND: Indomethacin
FBS: Fetal bovine serum
PBS: Phosphate-buffered saline
GAPDH: Glyceraldehyde-3-phosphate dehydrogenase
CIA: Collagen-induced arthritis
IL: Interleukin
Ctrl: Control
CRP: c-reactive protein
DAPI: 40,6-diamidino-2-phenylindole

## References

[1] Scott DL, Wolfe F, Huizinga TWJ. Rheumatoid arthritis. Lancet [Internet]. 2010;376:1094–108. http://www.sciencedirect.com/science/article/pii/S0140673610608264. 10.1016/S0140-6736(10)60826-4 20870100

[2] Branimir Anić MM (2014). Pathogenesis of rheumatoid arthritis. Reumatizam.

[3] Huber LC, Distler O, Tarner I, Gay RE, Gay S, Pap T (2006). Synovial fibroblasts: key players in rheumatoid arthritis. Rheumatology.

[4] Xu X, Chen H, Zhang Q, Xu J, Shi Q, Wang M. MiR-650 inhibits proliferation, migration and invasion of rheumatoid arthritis synovial fibroblasts by targeting AKT2. Biomed Pharmacother [Internet]. 2017;88:535–41. http://www.sciencedirect.com/science/article/pii/S0753332216319370. 10.1016/j.biopha.2017.01.063 28129626

[5] Zhang QR, Zhong ZF, Sang W, Xiong W, Tao HX, Zhao GD, et al. Comparative comprehension on the anti-rheumatic Chinese herbal medicine Siegesbeckiae Herba: Combined computational predictions and experimental investigations. J Ethnopharmacol [Internet]. 2019;228:200–9. http://www.sciencedirect.com/science/article/pii/S037887411830223X. 10.1016/j.jep.2018.09.023 30240786

[6] Hong YH, Weng LW, Chang CC, Hsu HF, Wang CP, Wang SW, et al. Anti-inflammatory effects of siegesbeckia orientalis ethanol extract in in vitro and in vivo models. Biomed Res Int. Hindawi Publishing Corporation; 2014;2014. 10.1155/2014/329712 PMC4160630 25328884

[7] Huh J-E, Baek Y-H, Lee J-D, Choi D-Y, Park D-S (2008). Therapeutic effect of *Siegesbeckia pubescens* on cartilage protection in a rabbit collagenase-induced model of osteoarthritis. J Pharmacol Sci..

[8] Jeon CM, Shin IS, Shin NR, Hong JM, Kwon OK, Kim HS (2014). Siegesbeckia glabrescens attenuates allergic airway inflammation in LPS-stimulated RAW 264.7 cells and OVA induced asthma murine model. Int Immunopharmacol.

[9] Kim H-M, Lee J-H, Won J-H, Park E-J, Chae H-J, Kim H-R (2001). Inhibitory effect on immunoglobulin E production in vivo and in vitro by Siegesbeckia glabrescens. Phyther Res.

[10] Cho YR, Choi SW, Seo DW (2013). The in vitro antitumor activity of Siegesbeckia glabrescens against ovarian cancer through suppression of receptor tyrosine kinase expression and the signaling pathways. Oncol Rep.

[11] Linghu K-G, Zhao GD, Xiong W, Sang W, Xiong SH, Tse AKW, et al. Comprehensive comparison on the anti-inflammatory effects of three species of Sigesbeckia plants based on NF-κB and MAPKs signal pathways in vitro. J Ethnopharmacol [Internet]. 2020;250:112530. http://www.sciencedirect.com/science/article/pii/S0378874119320197. 10.1016/j.jep.2019.112530 31883476

[12] Sims NA, Green JR, Glatt M, Schlict S, Martin TJ, Gillespie MT (2004). Targeting osteoclasts with zoledronic acid prevents bone destruction in collagen-induced arthritis. Arthritis Rheum.

[13] Linghu K-G, Wu G-P, Fu L-Y, Yang H, Li H-Z, Chen Y (2019). 1,8-Cineole Ameliorates LPS-Induced Vascular Endothelium Dysfunction in Mice via PPAR-γ Dependent Regulation of NF-κB. Front Pharmacol..

[14] Ray A, Kumar D, Shakya A, Brown CR, Cook JL, Ray BK (2004). Serum amyloid A-activating factor-1 (SAF-1) transgenic mice are prone to develop a severe form of inflammation-induced arthritis. J Immunol..

[15] Conigliaro P, Triggianese P, De Martino E, Chimenti MS, Sunzini F, Viola A, et al. Challenges in the treatment of rheumatoid arthritis. Autoimmun Rev [Internet]. 2019; http://www.sciencedirect.com/science/article/pii/S1568997219301107. 10.1016/j.autrev.2019.05.007 31059844

[16] Guo Q, Wang Y, Xu D, Nossent J, Pavlos NJ, Xu J. Rheumatoid arthritis: pathological mechanisms and modern pharmacologic therapies. Bone Res [Internet]. 2018;6:15. 10.1038/s41413-018-0016-9. 10.1038/s41413-018-0016-9 PMC5920070 29736302

[17] Lu Y, Xiao J, Wu Z-W, Wang Z-M, Hu J, Fu H-Z, et al. Kirenol exerts a potent anti-arthritic effect in collagen-induced arthritis by modifying the T cells balance. Phytomedicine [Internet]. 2012;19:882–9. http://www.sciencedirect.com/science/article/pii/S0944711312001535. 10.1016/j.phymed.2012.04.010 22673798

[18] Mortensen RF (2001). C-reactive protein, inflammation, and innate immunity. Immunol Res.

[19] Kowalski ML, Wolska A, Grzegorczyk J, Hilt J, Jarzebska M, Drobniewski M, et al. Increased responsiveness to Toll-like receptor 4 stimulation in peripheral blood mononuclear cells from patients with recent onset rheumatoid arthritis. Mediators Inflamm. 2008;2008. 10.1155/2008/132732 PMC2435281 18584044

[20] Khansai M, Phitak T, Klangjorhor J, Udomrak S, Fanhchaksai K, Pothacharoen P (2017). Effects of sesamin on primary human synovial fibroblasts and SW982 cell line induced by tumor necrosis factor-alpha as a synovitis-like model. BMC Complement Altern Med.

[21] Roman-Blas JA, Jimenez SA. NF-κB as a potential therapeutic target in osteoarthritis and rheumatoid arthritis. Osteoarthr Cartil [Internet]. 2006;14:839–48. http://www.sciencedirect.com/science/article/pii/S1063458406001105. 10.1016/j.joca.2006.04.008 16730463

[22] Zhong Z, Zhang Q, Tao H, Sang W, Cui L, Qiang W (2019). Anti-inflammatory activities of Sigesbeckia glabrescens Makino: combined in vitro and in silico investigations. Chin Med.

[23] Makarov SS (2001). NF-κB in rheumatoid arthritis: a pivotal regulator of inflammation, hyperplasia, and tissue destruction. Arthritis Res Ther.

[24] Han Z, Boyle DL, Manning AM, Firestein GS (1998). AP-1 and NF-kB regulation in rheumatoid arthritis and murine collagen-induced arthritis. Autoimmunity.

[25] Rosillo MÁ, Alarcón-De-La-Lastra C, Castejón ML, Montoya T, Cejudo-Guillén M, Sánchez-Hidalgo M (2019). Polyphenolic extract from extra virgin olive oil inhibits the inflammatory response in IL-1β-activated synovial fibroblasts. Br J Nutr.

